# Using Social Listening for Digital Public Health Surveillance of Human Papillomavirus Vaccine Misinformation Online: Exploratory Study

**DOI:** 10.2196/54000

**Published:** 2024-03-08

**Authors:** Dannell Boatman, Abby Starkey, Lori Acciavatti, Zachary Jarrett, Amy Allen, Stephenie Kennedy-Rea

**Affiliations:** 1 Department of Cancer Prevention & Control School of Medicine West Virginia University Morgantown, WV United States; 2 West Virginia University Cancer Institute Morgantown, WV United States

**Keywords:** human papillomavirus, HPV, vaccine, vaccines, vaccination, vaccinations, sexually transmitted infection, STI, sexually transmitted disease, STD, sexual transmission, sexually transmitted, social media, social listening, cancer, surveillance, health communication, misinformation, artificial intelligence, AI, infodemiology, infoveillance, oncology

## Abstract

Despite challenges related to the data quality, representativeness, and accuracy of artificial intelligence–driven tools, commercially available social listening platforms have many of the attributes needed to be used for digital public health surveillance of human papillomavirus vaccination misinformation in the online ecosystem.

## Introduction

The COVID-19 pandemic accelerated the spread of misinformation online, creating an “infodemic” that had profound effects on health behavior [1]. The breadth and depth of COVID-19 misinformation expanded to include all vaccinations, such as human papillomavirus (HPV) vaccination, depressing already suboptimal vaccination uptake in the United States [1,2]. As HPV vaccination is critical to the prevention of various cancers, this could pose significant cancer control challenges in the future [2]. There is an urgent need to address HPV vaccination misinformation to increase HPV vaccination uptake [2]. Behavioral interventions can counter misinformation online, but they are typically limited to a single social media platform without geographic specificity [3].

*Public health surveillance (PHS)* is defined by the Centers for Disease Control and Prevention (CDC) as “the ongoing, systematic collection, analysis, interpretation, and dissemination of data regarding a health-related event for use in public health action to reduce morbidity and mortality and to improve health” [4]. Digital PHS (DPHS) uses data from online sources, often collected outside of traditional PHS, for similar purposes [5]. There has been debate as to the ethics of using publicly available online data for DPHS [5]. However, the pandemic illustrated the need for user-friendly, timely, interactive digital tools to drive health-related intervention [6].

Social listening (SL) is the process of aggregating data from across online channels to collect real-time measures of emotions, opinions, and themes, typically through platform algorithms that rely on machine learning and artificial intelligence (AI) [7]. While SL platforms’ AI-driven tools for emotion and sentiment detection can be unreliable, machine learning provides an opportunity to “train” SL platforms for greater accuracy over time in the automated recognition of emotions and sentiments [8]. The World Health Organization Early AI-Supported Response With Social Listening Platform (WHO EARS) uses an SL dashboard to provide health professionals access to information from across the internet to assist in the development of timely responses to COVID-19 narratives that occur online at the global and country levels, highlighting the growing acceptance of such tools in public health [7].

The purpose of this exploratory study was to assess the feasibility of using a commercially available SL platform to monitor HPV vaccination misinformation online at the national (ie, within the United States overall) and state (ie, within Mississippi and Rhode Island) levels.

## Methods

### Ethical Considerations

This study received institutional review board exemption from West Virginia University (protocol #00152755).

### Study Design

Brandwatch was the commercially available SL platform selected for this exploratory study. It was selected after reviewing functionalities of leading SL platforms and having conversations about capabilities with representatives from Agorapulse, Brandwatch, Hootsuite, and Sprout Social. While most platforms had similar functionalities and data access, Brandwatch was selected based on opportunities to build queries with greater geographic specificity. While there is limited research on SL platform functionality within public health, Brandwatch was previously studied for the accuracy of AI-driven analyses [8]. The previously cited limitations of Brandwatch AI-driven tools informed the study team’s systematic, routine approach to training.

The research team received onboarding from Brandwatch through 5 structured, live training sessions. Two research team members completed a self-paced online training certificate. After onboarding was complete, the research team’s SL lead analyst (AS) built an HPV vaccination query within Brandwatch, using keywords and phrases identified through previous research and with research team consensus [9]. From this query, AS, with support from Brandwatch developers, created a dashboard to monitor online conversations within the United States overall and in 2 states—Mississippi, the US state with the lowest HPV vaccination rate, and Rhode Island, the US state with the highest vaccination rate. The research team regularly reviewed the query keywords and updated them as needed for increased relevancy and accuracy.

Brandwatch AI-driven tools were trained to recognize sentiments and emotions related to HPV vaccination. Sentiment categories for this study were different from the ones provided automatically by Brandwatch within the platform and were determined by the research team based on previous research [9]. Sentiment categories included “fact-based information,” “pro-vaccine opinions,” “misinformation,” “anti-vaccine opinions,” and “neutral comments.” These sentiment categories were built into the dashboard by a Brandwatch developer in conjunction with AS. The initial AI-driven recognition of these content categories was inaccurate. For example, all content that mentioned “cancer” was automatically considered negative by the SL platform AI. AS trained the AI-driven sentiment tool to recognize the intended content by reviewing aggregated social media comments, as well as other online articles and posts within Brandwatch, and adding them to the appropriate categories to spur AI recognition. During this AI training process, another sentiment category—“irrelevant”—was added, as content that used similar language but was not directly related to HPV was identified. The Brandwatch AI-driven sentiment tool was trained by AS routinely over a 6-month period to enhance the recognition of categories. This routine training significantly improved category recognition within the SL platform but was not completely accurate upon periodic spot reviews by the research team. The AI-driven tool for recognizing emotions automatically included categories such as “anger,” “disgust,” “fear,” “joy,” “sadness,” and “surprise.” Like the AI-driven sentiment tool, the identification of correct emotion categories was initially incorrect and required routine training by AS to improve accuracy.

Once the SL platform was built, the research team evaluated the dashboard, query, and implementation process notes to assess the feasibility of using a commercially available SL platform for HPV vaccination misinformation DPHS. This assessment was completed by using an adaptation of the CDC’s attributes for an effective PHS system [4]. The attributes adapted in this study were identified from CDC iterations published since 1988 [10]. The adaption of attributes involved the inclusion of consistent elements and associated definitions from across these CDC iterations; the addition of “cost” as a potential challenge to scaling; and the removal of “predictive value positive,” as the proposed DPHS approach would assess online narratives as opposed to a specific health condition. Consensus on each attribute was reached among the research team members.

## Results

Table 1 details each adapted PHS system attribute and the opportunities and limitations with regard to using a commercially available SL platform for HPV vaccination misinformation DPHS. Opportunities include user-friendly dashboards with real-time data monitoring and platform adaptability. For example, from June 21 to 24, 2023, the research team was able follow the spread of misinformation through social media posts related to a lawsuit filed by the Children’s Health Defense Fund, an organization led by prominent antivaccine activist Robert Kennedy Jr. However, while the SL platform dashboards are user-friendly, it took significant staff time, expertise, and routine maintenance to keep them relevant and as accurate as possible. Brandwatch was also found to be adaptable to the ever-changing online information ecosystem; however, the quality of this information was dependent on data access agreements with individual social media companies, which could change at any time. Additional challenges to using an SL platform for DPHS include concerns with data quality, representativeness, and the accuracy of AI-driven tools. There are limited ways to validate data within the SL platform itself. Data may be downloaded from Brandwatch and externally analyzed for sentiments and emotions, but this process would remove the AI-driven, automated nature of the SL platform and reduce the effectiveness of real-time monitoring in DPHS.

**Table 1:** Feasibility of using a commercial social listening platform for human papillomavirus vaccination misinformation digital public health surveillance. This was assessed based on attributes of public health surveillance systems adapted from the Centers of Disease Control and Prevention [4].

Attribute	Attribute description	Social listening opportunities	Social listening limitations
Usefulness	Contribution to prevention and control of misinformation	Events that may trigger misinformation spread can be identified in real time, providing an opportunity to target intervention	Unclear if targeted interventions can effectively shift online narratives
Simplicity	Simplicity of structure and ease of use	Dashboards can automate monitoring and provide easy-to-use tools to dig deeper into observable trends	Building effective queries requires a specialized skill set, including content area knowledge and experience with social media and online ecosystems
Flexibility	Adaptable to changing information and conditions	Queries can be adapted to new information and trends by changing keywords and phrases	Requires consistent monitoring by skilled personnel to ensure queries are reflective of current conditions
Data quality	Validity and completeness of data	Queries can include data beyond social media, providing a window into narratives in online public spaces	Data are limited by access provided by specific social media companies and the effectiveness of the query, along with a current lack of external data validation
Representativeness	Accurately describes flow of health information over time and distribution by place and person	Queries can monitor conversation trends over time, such as trends among audience panels and in various locations, which provide insights into demographics and geographic boundaries	Demographic and geographic information is imprecise and is limited based on availability
Timeliness	Lapse of time between misinformation and intervention	Conversations can be monitored in real time, providing opportunities for quick responses to misinformation	Lack of evidence-based responses to counter misinformation spread
Sensitivity	Ability to identify true cases and detect misinformation	Dashboard algorithms can be trained to detect changes in sentiments and emotions, providing an opportunity to respond to trends	Effectively training algorithms to detect sentiments and emotions is time-consuming and requires a specialized skill set
Stability	System is resilient to change	Can collect new sources of online data as they emerge to remain relevant in the shifting social media and online ecosystem	Changes to social media company policies can affect access to data sources
Acceptability	Willingness of persons and organizations to participate	Data collection is passive and does not burden participants with active data requests	Ethical concerns with online public data collection
Portability	Duplication of system in another setting	Social listening platforms can be purchased and adapted to different settings and health conditions, with no specialized hardware required for operation	Effectiveness of the queries may be limited by the personnel developing them and the sophistication of the selected social listening platform
Costs	Cost-effectiveness of the system	Online services can vary in price (≥US $2500 annually) based on the services needed for social listening	Sophisticated social listening platforms are more costly, although they provide greater access to data and tools

While Brandwatch was selected due to opportunities for greater geographic specificity, this functionality was limited in scope to only certain social media platforms, such as X (formerly Twitter). Furthermore, geographic specificity was limited based on whether social media users used geolocation functionalities and whether locations were mentioned in profiles or posts. Despite this, the research team identified and monitored different narratives in misinformation within the two states included in this exploratory study—Rhode Island and Mississippi—suggesting the potential importance of assessing online misinformation narratives based on geographic location. For example, on the same day in January 2024, the top trending story for Rhode Island focused on the Children’s Health Defense Fund lawsuit, while in Mississippi, the top story focused on childhood injury due to vaccination.

## Discussion

Our findings suggest that there are opportunities and challenges associated with using commercially available SL platforms to monitor HPV vaccination misinformation online at the national and state levels. While there were strengths across all PHS system attributes, there were also significant weaknesses. These weaknesses, particularly those related to data quality, representativeness, and the accuracy of AI-driven tools, reflect limitations to using current SL platforms for DPHS. If these challenges are addressed over time however, this level of DPHS could provide the foundation for different intervention opportunities, such as using skilled infodemiologists to counter online misinformation [11]. While the research team identified challenges with the accuracy of Brandwatch AI-driven tools, which matched previously published research [8], building DPHS capabilities now could provide critical infrastructure if and when such tools improve over time. If found to be effective in monitoring HPV vaccine misinformation, commercially available SL platforms may be adapted to other fields and health conditions. Findings may differ based on the SL platform used and vendor access agreements with social media companies. Future research should focus on increasing the specificity of geographic location, studying strategies to increase the accuracy of SL platform AI-driven tools, and testing targeted interventions using SL platforms.

## Abbreviations

AI: artificial intelligence
CDC: Centers for Disease Control and Prevention
DPHS: digital public health surveillance
HPV: human papillomavirus
PHS: public health surveillance
SL: social listening
WHO EARS: World Health Organization Early AI-Supported Response With Social Listening Platform
